# Dahuang Danshen Decoction Inhibits Pancreatic Fibrosis by Regulating Oxidative Stress and Endoplasmic Reticulum Stress

**DOI:** 10.1155/2021/6629729

**Published:** 2021-08-10

**Authors:** Xiaoqiang Liang, Mian Han, Xuelin Zhang, Xun Sun, Kui Yu, Congying Liu, Jiaqi Zhang, Cheng Hu, Jingzhe Zhang

**Affiliations:** ^1^Longhua Hospital Affiliated to Shanghai University of Traditional Chinese Medicine, Shanghai 200032, China; ^2^Experiment Center for Science and Technology, Shanghai University of Traditional Chinese Medicine, Shanghai 201203, China; ^3^Shanghai TCM-Integrated Institute of Vascular Anomalies, Shanghai TCM-Integrated Hospital, Shanghai University of Traditional Chinese Medicine, Shanghai, China

## Abstract

**Background:**

In Traditional Chinese Medicine (TCM), Dahuang Danshen decoction (DD) is used to treat pancreatic fibrosis. Pancreatic fibrosis is a typical manifestation of chronic pancreatitis (CP), which affects the digestive system. The therapeutic mechanisms of DD in pancreatic fibrosis are unclear.

**Aim:**

This study aimed to investigate the regulatory mechanisms of DD on oxidative stress and endoplasmic reticulum stress in CP.

**Materials and Methods:**

Experimental rats were intraperitoneally injected with 500 mg/kg BW of diethyldithiocarbamate (DDC) twice a week for six weeks to induce CP. At the same time, DD was administered orally at daily doses of 1.37 g/kg BW, 2.74 g/kg BW, and 5.48 g/kg BW to evaluate its treatment effects on CP. After all treatments, pancreatic tissues were harvested and subjected to H&E staining. Transmission electron microscopy (TEM) was also performed to show the endoplasmic reticulum structure in the pancreatic tissues. Immunohistochemistry was used to detect the *α*-SMA expression level in the pancreatic tissues. Metabolomics analysis of the serum and proteomics analysis of the pancreatic tissues were performed to reveal the changes of endogenous metabolites and proteins, respectively. Concentrations of GSH, MDA, SOD, ROS, col-1, and col-3 were determined using corresponding kits. The western blotting method was used to determine the protein levels of Keap-1, HO-1, NQO1, Nrf2, GRP, JNK, and caspase 12. The pancreatic mRNA levels of NQO1, GPX1, HO-1, GST-*π*, GRP, JNK, and caspase 12 were also determined by quantitative PCR. The interactions between TCM components and Keap-1 were investigated by molecular docking modeling.

**Results:**

The pathohistological results demonstrated that DD could ameliorate DDC-induced CP *in vivo*, indicated by reduction of *α*-SMA, col-1, col-3, TNF-*α,* and IL-6. DD increased serum levels of GSH and SOD but reduced pancreatic ROS. DD decreased cytoplasmic Keap-1 and increased Nrf2 nuclear localization. Correspondingly, DD increased the expression levels of Nrf2 downstream antioxidant genes NQO1, GPX1, HO-1, and GST-*π*. DD also decreased ERS hallmarks caspase 12 cleavage and GRP expression. Eventually, DD inhibited PSC activation by reducing JNK phosphorylation and MMK-3/p38 expression. Molecular docking analysis showed that salvianolic acid B and emodin had a good binding affinity toward Keap-1.

**Conclusions:**

These results demonstrated that DD could ameliorate the oxidative and endoplasmic reticulum stress through releasing Nrf2 from Keap-1 binding and inducing the downstream antioxidant enzymes. As a result, DD could thwart pancreatic fibrosis by inhibiting PSCs activation, which was induced by OS and ERS through JNK and MMK3/p38 pathways.

## 1. Introduction

Chronic pancreatitis (CP) is a common digestive system disease. Its typical clinical manifestations are steatorrhea, abdominal pain, weight loss, polyuria, and other pancreatic endocrine disorders [1]. Pancreatic fibrosis is also a typical feature of CP caused by various reasons [2, 3]. The associated pathological events comprise the recruitment of inflammatory cells, parenchymal atrophy, and excessive extracellular matrix (ECM) deposition in the pancreas' exocrine region [4]. The hallmark feature of pancreatic fibrosis is the deposition of ECM caused by the activation of pancreatic stellate cells (PSCs), which increases ECM synthesis [5, 6].

Inflammation plays an important role in the occurrence and development of pancreatic fibrosis. Recent studies have shown that some inflammation factors such as tumor necrosis factor-*α* (TNF-*α*) and interleukin-6 (IL-6) can induce OS. ERS usually happens together with or after OS, shown by the expression of its marker gene Glucose-regulated protein 78 (GRP78). OS induces PSCs activation through JNK and MMK3/p38 pathways, while ERS induces caspase 12 cleavage and thus increases apoptosis, which promotes PSCs activation [7, 8].

Some researches have demonstrated that therapies of preventing OS and ERS can effectively alleviate pancreatic tissue damage and pancreatic fibrosis degree in chronic pancreatitis [9]. Therefore, antioxidants and ER stress reduction are important targets for the treatment of pancreatic fibrosis. One such target is nuclear erythroid-related factor 2 (Nrf2). When dissociating from Keap-1 and translocating into nucleus, it drives ROS detoxification through induction of many antioxidant enzymes, such as superoxide dismutase (SOD), heme oxygenase (HO)-1, GST-*π*, and GPX1.

The Dahuang Danshen decoction (DD) is used to treat pancreatic fibrosis in Traditional Chinese Medicine (TCM) with appreciable therapeutic effects [10]. It is composed of *Rheum palmatum* L. stem and *Salvia miltiorrhiza* Bge., which promote blood circulation and curb blood stasis [10]. Our previous studies confirmed that DD could inhibit ECM synthesis, promote ECM degradation, and downregulate TGF-*β*1 expression to inhibit PSCs activation, thereby altering the course of pancreatic fibrosis [11]. This study was designed to shed further light on the therapeutic effect and mechanism of DD on DDC induced rat CP model based on proteomics and metabolomics study.

## 2. Materials and Methods

### 2.1. Reagents and Chemicals

The *Rheum palmatum* L. stem and *Salvia miltiorrhiza* Bge. were purchased from Longhua Hospital, Shanghai University of Traditional Chinese Medicine, China. Acetonitrile and methanol were purchased from Fisher Chemicals (Waltham USA). FASP Kits for col-1, col-3, ROS, MDA, SOD, and GSH were purchased from Nanjing Jiancheng Bioengineering Institute (Nanjing, China). Cytoplasmic extraction reagents and Pierce BCA Protein Assay Kit were purchased from Thermo Fisher Scientific (Waltham, MA). PrimeScript RT Master Mix and SYBR Premix Ex Taq were from TaKaRa (Shiga, Japan). Antibodies for immunoblotting, including anti-GAPDH, anti-Keap1, anti-HO-1, anti-Nrf2, anti-GRP, anti-caspase 12, anti-JNK, and anti-p38, were purchased from Cell Signaling Technology (Danvers, MA). Carboxymethyl cellulose sodium (CMC-Na) was obtained from Sigma Co. (St. Louis, MO).

### 2.2. Preparation of the Dahuang Danshen Decoction Extract

The DD decoction was prepared by soaking 137 g of *Rheum palmatum* L. stem and 137 g of *Salvia miltiorrhiza* Bge. in water (2 L) for 30 minutes. The mixture was concentrated to yield 500 mL of decoction. The concentration of raw herbs in the decoction was 0.548 g/mL.

### 2.3. Composition Analysis of the DD by UPLC-MS/MS

In this study, 1 mL of the DD was mixed with methanol in a 10 mL brown volumetric flask and ultrasonicated completely. The supernatant was used for composition analysis after centrifugation at 15000 g for 10 min and filtration through a 0.22 *μ*m membrane. A C_18_ column (100 mm × 2.1 mm, 1.8 *μ*m) was used for chromatographic separation. The mobile phase consisted of a mixture of water with 0.1% formic acid (A) and methanol (B). The elution conditions were as follows: 0–5 min, 90% A; 5–17 min, 90%-5% A; and 17–20 min, 5% A.

### 2.4. Experimental Animals and Experimental Design

According to the guidelines approved by the experimental animal ethical committee, male SD rats (200 ± 20 g) were housed in the laboratory animal center of Shanghai University of Traditional Chinese Medicine (Ethical license number: PZSHUTCM190927018). Fifty rats were randomly divided into five groups (*n* = 10) as follows: vehicle group, intraperitoneal injection of normal saline; DDC model, intraperitoneal injection of 500 mg/kg BW of DDC in saline twice a week for 6 weeks; and DDC + DD groups (1.37 g/kg BW, 2.74 g/kg BW, and 5.48 g/kg BW, respectively), DD orally administered with different dosages once a day for 6 weeks, starting at the same time with DDC treatment. The dosage in rats was calculated from the clinical adult dose. Animals were sacrificed 24 h after the last administration, and the plasma and pancreatic tissues were immediately collected for further analysis.

### 2.5. Histopathological Assessment

Half of the pancreatic tissues were fixed in 10% formalin, then embedded in paraffin, sliced, stained with hematoxylin and eosin (H&E) dyes, and assessed under a microscope. The other half of the pancreatic tissues were fixed in 2.5% glutaraldehyde stationary solution, followed by dehydration, embedding, and solidification. The pancreatic tissue's ultrastructural changes were observed under a projection electron microscope (FEI company, Tecnai G2 Spirit BioTWIN, USA).

### 2.6. ELISA Assay for Profibrotic and Proinflammatory Cytokines

Plasma was kept at room temperature and centrifuged at 860 g to collect serum. The serum concentrations of col-1, col-3, TNF-*α,* and IL-6 were determined by the ELISA method using their specific assay kits (Nanjing Jiancheng Bioengineering Institute).

### 2.7. Analysis of Serum GSH, MDA, SOD, and ROS in the Pancreas

Weighted pancreatic tissues were homogenized in normal saline (tissue: solvent = 1 : 9). The serum GSH, MDA, and SOD, as well as pancreatic ROS, were determined by specific assay kits according to the manufacturer's instructions.

### 2.8. RNA Extraction and Quantitative Real-Time PCR

Total RNA from the pancreatic tissues was extracted using a commercial extraction kit. Real-time PCR was performed using the SYBR Premium Ex Taq II kit (Takara, Japan) with primers from the Shanghai Haojia Gene Company (Shanghai, China). The experiment was performed in triplicate, and the obtained data were processed on an ABI QuantStudio 3.0. The negative control reaction was used to confirm the absence of contamination. We used the GAPDH to normalize all the mRNA expression levels.

### 2.9. Proteomics Study

For protein extraction and digestion, pancreatic tissue proteins were extracted and digested according to the commercial FFPE-FASPTM Protein Digestion Kit (Expedeon Inc., San Diego, CA). Sequence-level trypsin (Promega, Madison, MI) was added into the filter for digestion. The setup was incubated over night at 37°C, after which the peptides were collected by centrifugation at 15000 g for 10 min. Finally, the peptide sample was desalted using a ZipTip C18 tip (Millipore, Billerica, MA) before analysis.

In nano-LC-MS/MS and data analysis, for proteomic analysis, the peptides were separated using an acclaim PepMap RSLC C18 column (75 *μ*m × 25 cm, 2 *μ*m, nanoViper, 100 A) on a nanoflow HPLC Easy-nLC 1000 system (Thermo Fisher Scientific). The mobile phase consisted of a mixture of water and 0.1% formic acid (A) and acetonitrile with 0.1% formic acid (B). The gradient was set as follows: 0–2 min, 2–8% B; 2–112 min, 8–28% B; 112–114 min, 28–90% B; and 114–120 min, 90% B.

Proteomic analyses were performed on an Orbitrap Fusion Lumos Mass Spectrometer (Thermo Fisher Scientific). The spray voltage was set at 2,100 V in positive ion mode. For the MS1 full scan, ions with m/z ranging from 350 to 1600 were acquire by Orbitrap Mass Analyzer at a resolution of 120,000. Precursor ions were selected and fragmented with higher collision dissociation (HCD) with normalized collision energy of 30%. Isolation window was set at 0.7 m/z. The collected spectral data were processed and analyzed by Proteome Discoverer 2.4 SP1 software (Thermo Fisher Scientific).

### 2.10. Serum Metabolites in DDC-Induced CP in Experimental Rats

For sample preparation for metabolomics study, serum (50 *μ*L) was thoroughly mixed with methanol (200 *μ*L, containing 30 *μ*g/mL chlorophenylalanine, internal standards). Then, the sample was centrifuged at 12000 r/min at 4°C for 15 min. The obtained supernatant was used for metabolomics study.

For UPLC-MS condition and data analysis, the serum metabolites profiling was performed on Ultimate 3000 UPLC system (Thermo Fisher Scientific) coupled with an Orbitrap Elite Mass Spectrometer (Thermo Fisher Scientific).

The metabolites were chromatographically separated on a Hss T3 column (100 mm × 2.1 mm, 1.8 *μ*m, ACQUITY UPLC) at a flow rate of 0.3 mL/min for 17 min. Buffer A consisted of 0.1% formic acid in water and buffer B consisted of 0.1% formic acid in acetonitrile. The gradient was set as follows: 0–2 min, 95% A; 2–12 min, 5% A; 12–15 min, 5% A; and 15–17 min, 95% A.

For metabolite identification and pathway analysis, the collected data were processed by Compound Discover 2.0 (Thermo Fisher Scientific) to identify potential biomarkers according to the online database (HMDB, KEGG, m/z cloud). The preprocessed data was imported into SIMCA-P software (Umetrics, Umea, Sweden) for principal component analysis (PCA) and orthogonal partial least squares discriminant analysis (OPLS-DA). MetaboAnalyst 4.0 was used for pathway analysis.

### 2.11. Molecular Docking

ChemBioDraw Ultra 17.0 to was used to draw the structure of the compounds (emodin and salvianolic acid B), and ChemBio3D Ultra 17.0 was used to create three-dimensional structures and MMFF94 for force field optimization. The three-dimensional structure of Keap-1 (PDB ID was 4IQK) was downloaded from the RCSB Protein Data Bank (http://www.rcsb.org/). Keap-1 and the compounds (Emodin and salvianolic acid B) were converted to the PDBQT format using Autodock Tools 1.5.6. Autodock vina 1.1.2 was applied for molecular docking studies [12, 13]. The accuracy of the calculation was increased by setting the parameter exhaustiveness to 20. Except specifically mentioned, other parameters are used as default values. The conformation with the highest score was selected, and the Free Maestro 11.9 was used to analyze the results.

### 2.12. Western Blotting

Cytosolic and nuclear proteins in tissues were isolated as described in NE-PER™ Nuclear and Cytoplasmic Extraction Reagents. Protein concentration was determined using a BCA protein assay kit. The total protein from pancreatic tissues was isolated by SDS-PAGE and transferred to a nitrocellulose membrane. Membranes were blocked with 2% bovine serum albumin (BSA) and then incubated with primary antibodies overnight at 4°C. After that, membranes were incubated with secondary antibodies for 1 h at room temperature and washed with TBST. The protein expressions were detected by staining with the TANON-2008 Gel imaging system, and the expression levels were analyzed by the optical density value of the ratio of target protein/GAPDH.

### 2.13. Statistical Analysis

All the results were expressed as mean ± SD for each group. For multiple comparisons, one-way ANOVA followed by Tukey's test was performed. *P* values less than 0.05 were considered statistically significant.

## 3. Results

### 3.1. Identification of the DD Decoction Constituents

UPLC-LTQ-Orbitrap/MS was used to identify the components of DD decoction. The total ion chromatogram was shown in Figure S1. A total of 86 compounds, including emodin, salvianolic acid, and danshensu were identified based on their accurate masses and MS/MS fragmentation patterns (Table 1).

### 3.2. Decoction of DD Attenuated DDC-Induced CP in Rats

**Figure 1:**
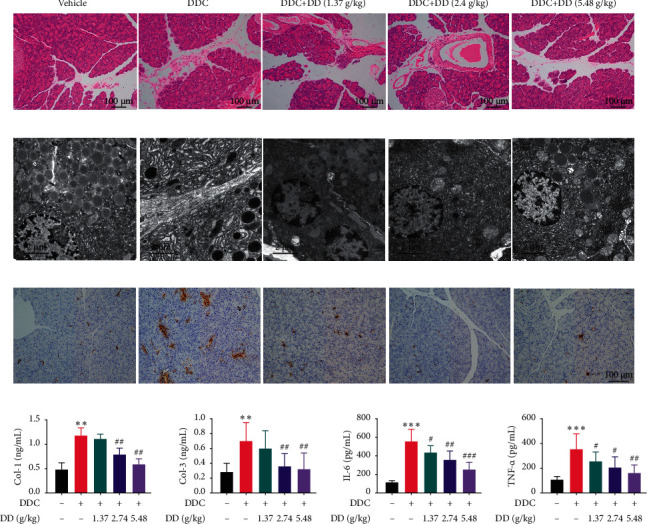
The decoction of DD ameliorated DDC-induced acute pancreatic injury *in vivo.* (a) H&E staining of rat pancreatic sections (×100, 200); (b) TEM of rat pancreas (×6000); (c) immunohistochemistry detected the pancreas *α*-SMA expression level; (d) Col-1 concentration in rat serum; (e) Col-3 concentration in rat serum; (f) serum protein concentrations of IL-6; (g) serum protein concentrations of TNF-*α*. Data are expressed as mean ± SD (*n* = 10; ^*∗∗*^*p* < 0.01 and ^*∗∗∗*^*p* < 0.001 compared to vehicle; ^#^*p* < 0.05, ^##^*p* < 0.01, and ^###^*p* < 0.001 compared to DDC).

In the vehicle treated rats, no obvious pathological changes of the pancreas were seen under a light microscope. The pancreas was clearly structured, the ducts were not significantly expanded, and the acinar arrangement was regular. A small number of fibrous connections were observed among the lobules. The acini and lobules were intact, some pancreatic stroma had mild edema, and inflammatory cells were rare. Compared to the vehicle group, the pancreatic tissue of the DDC treated rats had profound edema. The interlobular space of the pancreas was significantly enlarged. Some of the acinar cytoplasm and nucleus were concentrated and disintegrated, and some lobules formed a red-stained unstructured area, which was replaced by fibers. Notably, DD (1.37 g/Kg BW, 2.74 g/Kg BW, and 5.48 g/Kg BW) significantly reduced the pancreatic tissue edema and fibrosis in the pancreatic lobular compartments compared to the DDC group (Figure 1(a)).

Besides, in the vehicle group, the TEM demonstrated that only a few fibroblasts were found in the pancreas, and the endoplasmic reticulum was distributed uniformly in the cells without expansion. After DDC-induced chronic pancreatitis, there were a large amount of collagen deposited and obvious endoplasmic reticulum swelling compared to the vehicle group. Treatment with DD (1.37 g/Kg BW, 2.74 g/Kg BW, and 5.48 g/Kg BW) decoction showed normal cell morphology and relatively evenly distributed endoplasmic reticulum or slightly expanded (Figure 1(b)). These results indicated that DD inhibited pancreatic fibrosis by ameliorating the endoplasmic reticulum stress.

### 3.3. Decoction of DD Ameliorated DDC-Induced Pancreatic Inflammation in Rats

The *α*-SMA was analyzed to evaluate fibrosis. Col-1 and col-3 are also important indicators for evaluating fibrosis, which are involved in fibrosis development [14]. IL-6 and TNF-*α* were identified as crucial cytokines involved in the inflammatory process. In the present study, pancreatic *α*-SMA and the serum levels of col-1, col-3, IL-6, and TNF-*α* were significantly higher in the DDC-induced pancreatic model group than in the vehicle group. However, DD markedly reduced them in a dose-dependent manner (Figures 1(c)–1(g)).

### 3.4. Pathways Affected by DD

**Figure 2:**
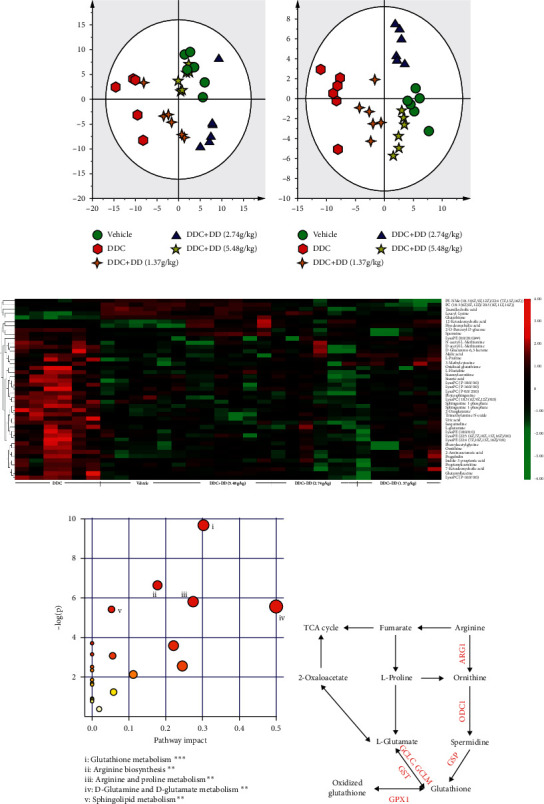
Serum metabolomics analysis in DDC-induced pancreatic injury in rats and pathway analysis. (a) PCA in negative mode. (b) PCA in positive mode. (c) Heatmap of significant changed serum metabolites between DDC and vehicle group (*n* = 6). (d) Pathway enrichment analysis. (e) The main influenced metabolic pathway.

The PCA and OPLS-DA are commonly used effective supervised multivariate analyses [15]. To explore how DD regulates DDC-induced alterations in rat serum, PCA was performed on the metabolomics data of all groups (Figures 2(a) and 2(b)). In both negative and positive ion modes, the plots of vehicle group were separated from those of DDC model groups, showing high discrimination. However, the plots of DDC + DD (5.48 g/kg) group were close to those of the vehicle group, indicating the similarity in metabolites of the two groups.

Heatmap of the differential metabolites demonstrated that DD treated groups had similar changing trends with the vehicle group but had an opposite changing trend with the DDC-induced model group, indicating that DD could regulate the metabolic pathways of DDC-induced CP rats (Figure 2(c)). Furthermore, these differential metabolic pathways involved glutathione metabolism, arginine biosynthesis, arginine and proline metabolism, D-glutamine and D-glutamate metabolism, and sphingolipid metabolism (Figure 2(d)). DD mainly regulated glutathione metabolism and arginine biosynthesis (Figure 2(e)). Besides, the TCA cycle was also involved in CP process as shown in Figure 2(e).

### 3.5. Analysis of the Differentially Expressed Proteins (DEPs)

**Figure 3:**
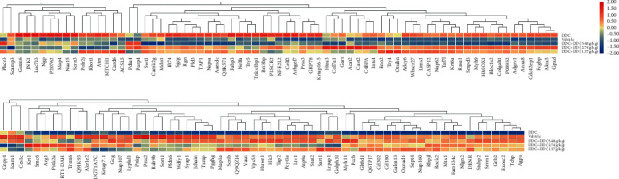
Heatmap of significant difference proteins (*n* = 6).

In this study, the DEPs were investigated by quantitative proteomics detection [16, 17]. The results showed that the expression levels of JNK and GRP were significantly elevated in the DDC group than in the vehicle group, and their expressions were downregulated by DD decoction. In contrast, the expression levels of Nrf2 and HO-1 were decreased in the DDC group and upregulated by the DD decoction (Figure 3).

### 3.6. Protein Interaction Networks of DD-Affected DEPs

We performed STRING to analyze the interactions between DEPs that showed functional interactions (Figure S2). A protein-protein interaction (PPI) network may integrate and analyze the known PPIs using proteomic and gene data. In the network, Mapk8, Arhgef7, GRP, and Polr2j were associated with other DEPs in PPI, which formed a large network associated with CP. Of these, GRP was related to Kng1, Gcg, and Eno2. A previous study showed that reactivation of Gcg expression in the pancreas has a glucoregulatory effect on the production of islet glucagon or GLP-1 [18].

### 3.7. Molecular Docking

**Figure 4:**
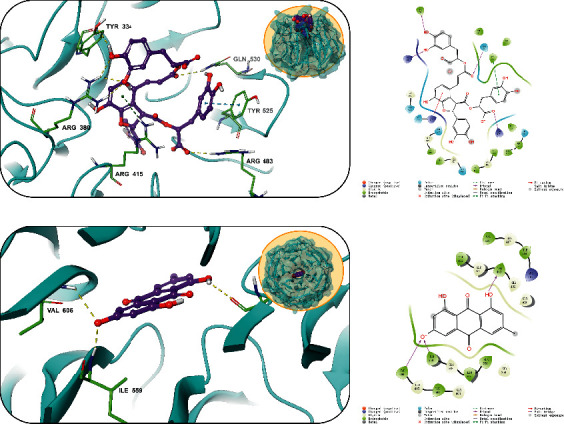
Molecular docking simulation of the main constituents of DD decoction and Keap-1. The docking model of salvianolic acid B in the binding site of Keap-1. (a) Three-dimensional molecular docking analysis. (b) Two-dimensional molecular docking analysis. The docking model of emodin in the binding site of Keap-1. (c) Three-dimensional molecular docking analysis. (d) Two-dimensional molecular docking analysis.

Molecular docking was performed to gain insights into the binding mode and affinities of the active compounds, namely, salvianolic acid B and emodin, toward the potential target at the molecular level. We docked emodin and salvianolic acid B into the active pocket of Keap-1, and the binding affinities of the two complexes were −8.5 and −10.2 kcal/mol, respectively (Figure 4). Hydrogen bonding contributes the most in stabilizing the Keap-1/emodin and Keap-1/salvianolic acid B complexes.

The interactions indicated that Keap-1 formed a stable complex with emodin and salvianolic acid B. Therefore, molecular docking studies offer a reasonable explanation for the interactions between Keap-1 and the compounds (emodin and salvianolic acid B).

### 3.8. The Decoction of DD Attenuated Pancreatic Oxidative Stress by Regulating the Keap-1 Pathway

**Figure 5:**
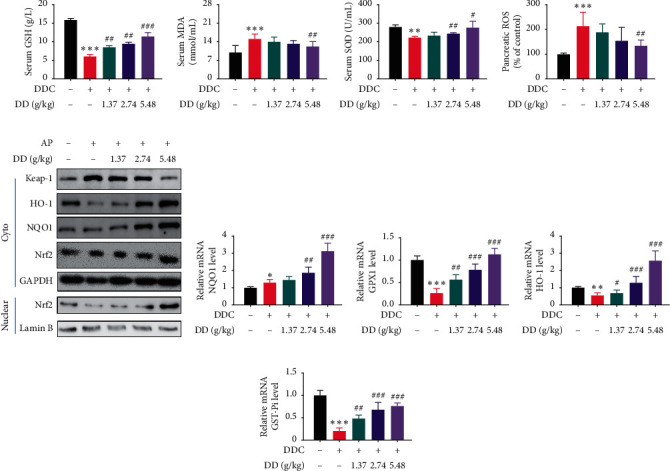
Decoction of DD ameliorated DDC-induced oxidative stress injury by regulating the Keap-1 pathway *in vivo*. (a) GSH content in serum. (b) MDA content in serum. (c) SOD in serum. (d) Pancreatic ROS levels were measured. (e) Pancreatic levels of Keap-1, HO-1, NQO1, and Nrf2 proteins were determined by WB. (f) Pancreatic mRNA level of NQO1. (g) Pancreatic mRNA level of GPX1. (h) Pancreatic mRNA level of HO-1. (i) Pancreatic mRNA level of GST-*π*. Data in (f)–(i) were determined by qRT-PCR. Data are expressed as mean ± SD (*n* = 10; ^*∗*^*p* < 0.05, ^*∗∗*^*p* < 0.01, and ^*∗∗∗*^*p* < 0.001 compared to vehicle; ^#^*p* < 0.05, ^##^*p* < 0.01, and ^###^*p* < 0.001 compared to CP).

In DDC group compared to the vehicle group, the serum levels of MDA and pancreatic ROS were significantly increased, while the serum levels of GSH and SOD were significantly reduced (*P* < 0.05). However, DD could reverse these trends in a dose-dependent way (Figures 5(a)−5(d)).

Moreover, the effect of DD on the pancreatic Keap-1/Nrf2 pathway was evaluated by PCR and western blot analyses (Figures 5(e)−5(i)). Our results demonstrated that Keap-1 was significantly increased in the DDC group and orally administered DD (1.37 g/kg BW, 2.74 g/kg BW, and 5.48 g/kg BW) significantly reduced the expression of Keap-1 compared to the model group (*P* < 0.05). The cytoplasm expression of Nrf2 remained unchanged, but its nuclear content decreased in DDC group and increased significantly after DD treatment. Nrf2, when dissociated from Keap-1, translocated into nucleus and inhibited ROS through induction of GST, GPX1, NQO1, and HO-1 genes. The expressions of HO-1, GPX1, and GST-*π* were downregulated in DDC group compared to the vehicle group and significantly increased in DD (1.37 g/kg BW, 2.74 g/kg BW, and 5.48 g/kg BW) groups compared to DDC group. NQO-1 expression was not significantly decreased in DDC group, but the increase in DD groups compared to DDC group was dramatic. These results strongly suggested that DD exerts its protective effects against DDC-induced oxidative stress injury in CP by regulating the Keap-1/Nrf2 pathway.

### 3.9. DD Decoction Ameliorated DDC-Induced Oxidative Stress and Endoplasmic Reticulum Stress

**Figure 6:**
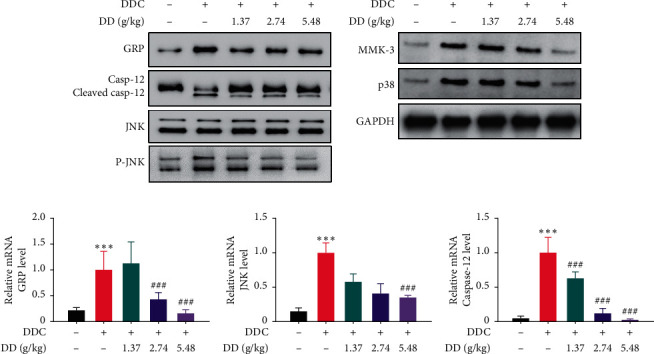
DD decoction ameliorated DDC-induced endoplasmic reticulum stress injury. (a) Pancreatic protein levels of GRP, Casp-12, JNK, p-JNK, MMK3, p38, and P-p38 were determined by western blot. (b) Pancreatic mRNA level of GRP. (c) Pancreatic mRNA level of JNK. (d) Pancreatic mRNA level of caspase 12.

OS induces PSCs activation through JNK or MMK-3/p38 pathways. Our *in vivo* study showed significantly increased pancreatic levels of MMK-3 and p38 in the DDC-induced CP rat model compared to the vehicle group. On the contrary, treatment with DD (1.37 g/kg BW, 2.74 g/kg BW, and 5.48 g/kg) resulted in the effective downregulation of these proteins compared to the CP rat model. Phosphorylated JNK was also downregulated in DDC group. Endoplasmic reticulum stress (ERS) usually occurs with or after OS and induces apoptosis. Apoptosis in the pancreas can also induce PSCs activation. GRP78 is the marker of ERS, and caspase 12 cleavage also happens during ERS to induce apoptosis. We also observed an increase in GRP78 and caspase 12 cleavage in the DDC group, while they were decreased after DD treatment (Figures 6(a)–6(d)). These crucial findings indicated that the protective effects against PSCs activation of DD might be through inhibition of OS and ERS.

## 4. Discussion

Nowadays, chronic pancreatitis (CP) is still a poorly understood disease [19]. Pancreatic fibrosis is a typical pathological manifestation of CP. The essence of pancreatic fibrosis is extracellular matrix (ECM) deposition which is caused by increased synthesis or reduced degradation of ECM [20, 21]. ECM is mainly synthesized by activated pancreatic stellate cells (PSCs) [22]. So, PSCs activation plays a key role in the fibrogenesis-associated CP and is considered to be the initiating event of pancreatic fibrosis [23]. PSCs activation is regulated by a variety of autocrine and paracrine cytokines, such as transforming growth factor-*β* (TGF-*β*), tumor necrosis factor-*α* (TNF-*α*), and interleukin-6 (IL-6) [24]. In detail, when CP-induced pancreatic injury existed, PSCs were stimulated from quiescent state to activated state to initiate pancreatic fibrosis. The *α*-SMA protein is involved in pancreatic fibrosis and is the sign of pancreatic fibrosis formation [25]. Diethyldithiocarbamate- (DDC-) induced CP is mainly associated with pancreatic inflammation and oxidative stress that lead to pancreatic fibrosis. In our study, we found that Dahuang Danshen decoction (DD) could ameliorate DDC-induced CP and pancreatic inflammation in rats. DD could reduce the pathological changes of pancreatic tissue and the expression of *α*-SMA. DD could also reduce the levels of col-1, col-3, IL-6, and TNF-*α* in serum.

Proteomics and metabolomics are widely used to discover new targets and signaling pathways in pharmacological researches. The results of both omics showed that the pathways related to oxidative stress and endoplasmic reticulum stress were significantly changed in DDC model. We confirmed that the expression changes of GST, HO-1, Nrf2, GRP78, and other related proteins were consistent with the proteomics results. In addition, we also found that ACSL5, Lyplal1, and ALOX5 proteins, which were related to lipid peroxidation, were significantly increased in DDC model. It indicated that there were a lot of lipid abnormalities in DDC model, which required further study.

Oxidative stress caused by the imbalance of oxidation and antioxidation in the body is harmful, which produces a large number of oxidation intermediates. It is reported that oxidative stress is involved in the occurrence and development of pancreatic fibrosis [26, 27]. It can promote the activation of PSCs through activation of JNK and MMK3/p38 pathways and thus aggravate the process of pancreatic fibrosis [28]. Endoplasmic reticulum stress (ERS) is a pathological process of subcellular organelles in the early stages of multiple stress events and is a common pathway of nuclear stress and mitochondrial stress [27]. ERS is a self-protective mechanism of cells, which protects the endoplasmic reticulum against protein misfolding or unfolding, restores the endoplasmic reticulum's homeostasis, improves the intercellular environment, and maintains cell viability and normal cell function. However, when the protective objectives are not achieved, ERS induces apoptosis. A previous study showed that ERS-induced apoptosis is an important factor leading to fibrosis [30]. GRP is a protein marker for the ERS response [31]. The caspase 12 pathway is a unique apoptotic pathway of the endoplasmic reticulum stress. In our DDC model rats, OS and ERS were increased, indicated by increased pancreatic ROS and other OS markers and increased GRP78 and caspase 12 cleavage, respectively. After DD treatment, these markers were reduced. Therefore, reducing OS, as well as ERS, is essential in the protective effect of DD against PSCs activation and pancreatic fibrosis.

Nuclear erythroid-related factor 2 (Nrf2) is the most critical oxidative stress-regulating nuclear transcription factor, which is usually coupled with its negative regulatory protein Keap-1 and immobilized in the cytoplasm in an inactive state [32]. When Nrf 2 is released from Keap-1, it translocates into the nucleus, binds to the response elements of antioxidant target genes, such as SOD, HO-1, NQO-1, GST-*π*, and GPX1, and therefore induces their expression. Although OS itself actually stimulates this process to form a negative response loop, when the ROS was severely increased by exogenous stimulus, such as DDC in our study, the negative response loop was disturbed by highly elevated Keap-1, which resulted in an inhibition of antioxidant enzymes expression. That was the major cause of elevated OS and ERS by DDC. Among the major components we identified in DD decoction, salvianolic acid B and emodin could be docked into the active pocket of Keap-1 protein to compete with Nrf2. Therefore, upon DD treatment, Nrf2 was dissociated from Keap-1 and highly activated those antioxidant enzymes. In this way, OS and ERS were relieved, and their activation of PSCs were reduced.

## 5. Conclusion

Our study demonstrated the promising protective effects of DD against DDC-induced CP fibrosis by regulating inflammatory factors, as well as the oxidative and endoplasmic reticulum stress. By releasing Nrf2 from Keap-1 in the cytoplasm, DD could increase the expression of Nrf2 downstream genes, including NQO1, HO-1, GPX1, GST-*π,* and SOD. Therefore, oxidative stress and subsequent ERS were reduced, which were indicated by reduced level of GSH and GRP78, as well as caspase 12 cleavage. These changes resulted in inhibition of PSCs activation through reduction of JNK and MMK3/p38 signaling.

## Figures and Tables

**Table 1:** Identified compounds in DD decotion.

No.	Name	Exact mass	RT (min)	Mode
1	3-[(carboxycarbonyl)amino]-L-alanine	175.9636	0.723	−
2	L-Glutamic acid	147.0528	0.866	+
3	Betaine	117.0785	0.894	+
4	Quinic acid	192.0633	0.932	−
5	Cytosine	111.0429	0.948	+
6	2-Pyrrolidinecarboxylic acid	115.0629	0.984	+
7	3-Furfuryl 2-pyrrolecarboxylate	191.0583	1.235	+
8	Citric acid	192.0271	1.263	−
9	Nicotinamide	122.0478	1.265	+
10	p-Coumaric acid	164.0472	1.391	+
11	Adenosine	267.0969	1.412	+
12	Acetophenone	120.0573	1.478	+
13	Maleic acid	116.0114	1.515	−
14	Guanosine	283.0917	1.546	+
15	Isoguanosine	283.0914	1.563	−
16	Pyrogallol	126.032	1.758	−
17	L-Phenylalanine	165.079	2.643	+
18	Shikimic acid	174.053	2.799	−
19	Gallic acid	170.0216	3.34	−
20	Caffeic acid	180.0425	3.403	−
21	(R)-Mandelic acid	152.0474	3.416	−
22	Protocatechuic acid	154.0269	3.729	−
23	Gallocatechin	306.0741	3.771	+
24	3,4-Dihydroxyphenylethanol	154.0633	3.956	−
25	5-Acetylsalicylic acid	180.0424	4.31	−
26	Catechin hydrate	290.0783	4.331	+
27	Cianidanol	290.0783	4.444	+
28	Procyanidin B2	578.1417	4.559	+
29	Apigenin-7-O-*β*-D-glucoside	432.1052	4.685	+
30	4-Hydroxybenzoic acid	138.0321	4.89	−
31	p-Hydroxybenzaldehyde	122.0364	4.904	+
32	Salicylic acid	138.0329	4.959	−
33	Isoferulic acid	194.0579	5.024	−
34	Procyanidin B1	578.1417	5.083	+
35	Epicatechin	290.0783	5.093	+
36	Protocatechualdehyde	138.0314	5.343	+
37	Ferulic acid	194.0579	5.451	−
38	Chrysophanol 8-O-*β*-D-glucoside	416.1106	5.492	+
39	4-Methylumbelliferone	176.0474	5.5	+
40	Polydatin	390.1314	5.604	+
41	Resveratrol	228.0786	5.611	+
42	Rosmarinic acid	360.0836	5.714	−
43	Iridin	522.1364	5.885	−
44	7-Hydroxycoumarin	162.0316	5.892	+
45	p-Hydroxy-cinnamic acid	164.0477	6.026	−
46	Diosmetin-7-O-*β*-D-glucopyranoside	462.1157	6.051	+
47	Isobergapten	216.042	6.071	+
48	Antrapurol	240.0421	6.071	−
49	Naringenin	272.0686	6.132	+
50	Benzoic acid	122.0364	6.139	+
51	Phloretin	274.0841	6.218	−
52	Cinnamic acid	148.0526	6.256	−
53	Lithospermic acid	538.1098	6.406	+
54	5,7-Dihydroxychromone	178.0264	6.752	+
55	Danshensu	198.0526	6.936	−
56	Salvianolic acid C	492.1047	6.957	+
57	Isoimperatorin	270.0892	7.009	−
58	7-Methoxy-4-methylcoumarin	190.0627	7.264	+
59	Salvianolic acid A	494.1195	7.27	−
60	Emodin	270.0525	7.345	−
61	Hydroxygenkwanin	300.0632	7.428	−
62	Noreugenin	192.0422	7.437	+
63	Bergaptol	194.0579	7.514	+
64	Isoliquiritigenin	256.0732	7.574	+
65	Genistein	270.0525	7.634	+
66	Morin	302.0425	7.679	+
67	Fisetin	286.0476	7.707	+
68	Daidzein	254.0578	7.76	+
69	Liquiritigenin	256.0732	7.798	+
70	Indigo	262.0743	7.848	+
71	Coumarin	146.0367	8.074	+
72	Aloe emodin	270.0525	8.203	−
73	Baicalein	270.0525	8.204	+
74	Emodin-3-methyl ether/physcion	284.0684	8.262	+
75	Naringenin chalcone	272.0686	8.28	+
76	Kaempferol	286.0476	8.568	+
77	Oroxylin A	284.0684	8.648	+
78	Paeonol	166.063	9.123	+
79	Ethyl ferulate	222.0891	9.156	−
80	Decursinol	246.0893	9.434	+
81	Rheic acid	284.0316	9.683	−
82	Rubiadin	254.0577	9.857	−
83	Dihydrotanshinone I	278.0944	11.348	+
84	Cryptotanshinone	296.141	12.438	+
85	Calycosin	284.0684	12.939	+
86	Tanshinone IIA	294.1256	13.459	+

## Data Availability

The data used to support the findings of this study are included within the supplementary information files.

## Abbreviations

DD: Dahuang Danshen
DDC: Diethyldithiocarbamate
CP: Chronic pancreatitis
LC-MS: Liquid chromatography mass spectrometry
ROS: Reactive oxygen species
GSH: Glutathione
MDA: Malondialdehyde
GST: Glutathione-S-transferase
ECM: Excessive extracellular matrix
PSC: Pancreatic stellate cells
PCA: Principal component analysis
OPLS-DA: Orthogonal partial least squares discriminant analysis
SOD: Superoxide dismutase
PPI: Protein-protein interaction
TGF-*β*: Transforming growth factor-*β*
PDGF: Platelet-derived growth factor
TNF-*α*: Tumor necrosis factor-*α*
IL-1: Interleukin-1
IL-6: Interleukin-6
ERS: Endoplasmic reticulum stress.

## Supplementary Materials

Supplementary Materials Figure S1: UPLC-MS/MS analysis of DD decoction in positive ion mode (a) and negative ion mode (b). Figure S2: A STRING interaction network diagram enriched by significantly different proteins.
