# Supplementary material for: Dahuang Danshen Decoction Inhibits Pancreatic Fibrosis by Regulating Oxidative Stress and Endoplasmic Reticulum Stress
Source: Evid Based Complement Alternat Med. 2021 Aug 10;2021:6629729. doi: 10.1155/2021/6629729 (PMC8371665; doi:10.1155/2021/6629729)
Supplement: Supplementary Materials — Figure S1: UPLC-MS/MS analysis of DD decoction in positive ion mode (a) and negative ion mode (b). Figure S2: A STRING interaction network diagram enriched by significantly different proteins. [file 6629729.f1.docx]

Supplemental information：

**
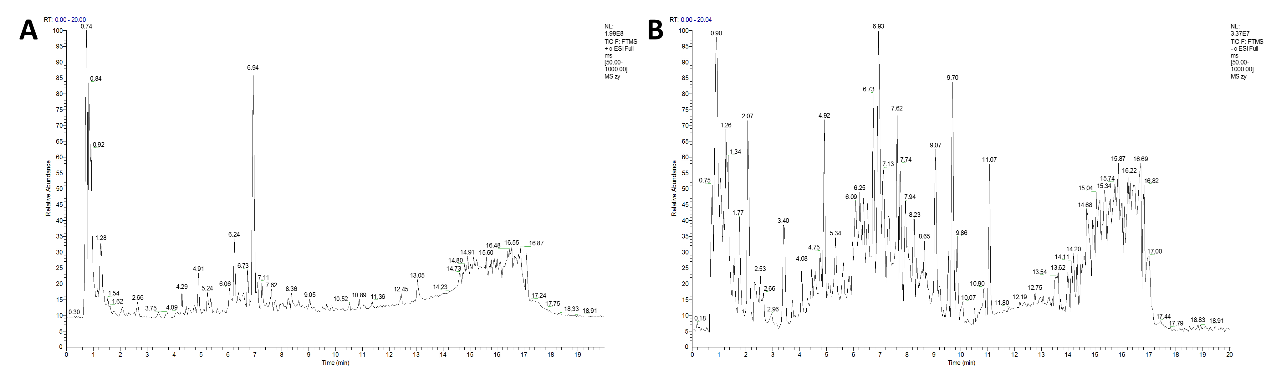
**

Fig.S1 UPLC-MS/MS analysis of DD decoction in positive ion mode (A) and negative ion mode (B)


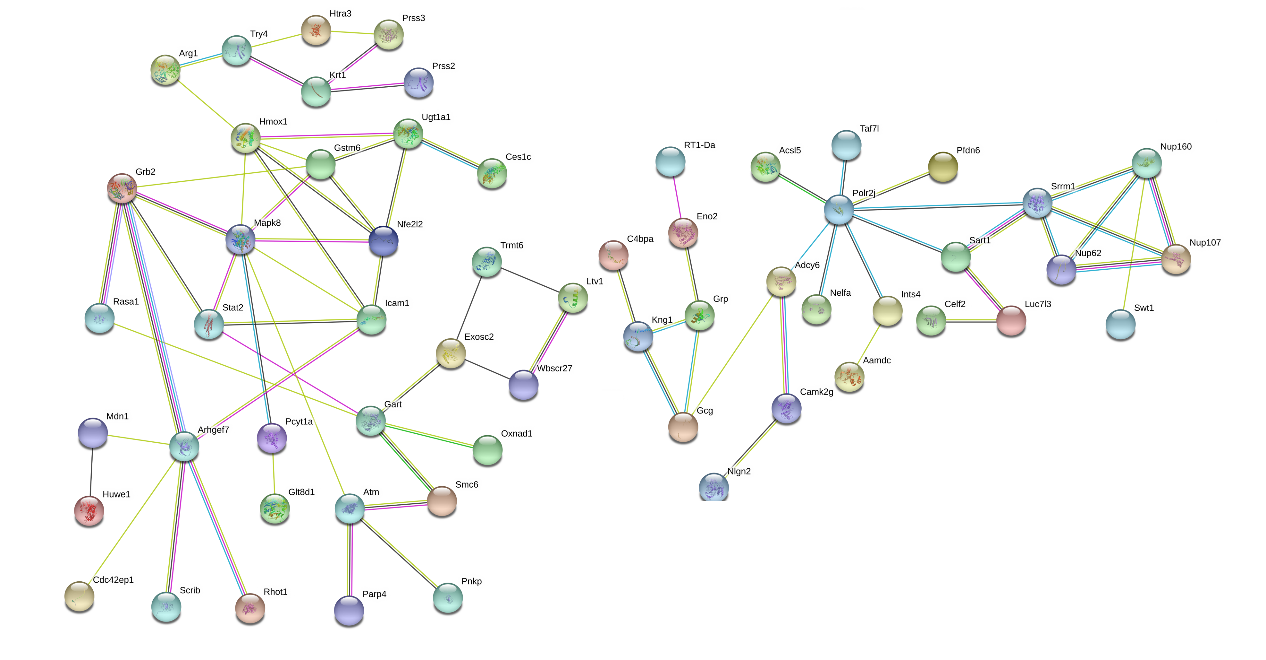


Fig.S2 A STRING interaction network diagram enriched by significantly different proteins.
